# Relationship Between Liver Steatosis, Pancreas Steatosis, Metabolic Comorbidities, and Subclinical Vascular Markers in Children with Obesity: An Imaging-Based Study

**DOI:** 10.3390/jcm14197048

**Published:** 2025-10-06

**Authors:** Kenza El Ghomari, Anna Voia, Jean-Baptiste Moretti, Anik Cloutier, Guy Cloutier, Ramy El Jalbout

**Affiliations:** 1 Department of Radiology, Faculty of Medicine, Université de Montréal, Montreal, QC H3C3J7, Canada; 2 Centre de Recherche du Centre Hospitalier Universitaire Sainte-Justine (CRCHUSJ), Montreal, QC H3T1C5, Canada; 3 Centre de Recherche du Centre Hospitalier de L’Université de Montréal (CRCHUM), Montreal, QC H2X0A9, Canada

**Keywords:** liver steatosis, pancreas steatosis, children, intima-media thickness, NIVE, MRI, MASLD

## Abstract

**Background**: Metabolic dysfunction-associated steatotic liver disease (MASLD) is prevalent in adolescents with obesity and is linked to insulin resistance and cardiovascular disease (CVD). Pancreas steatosis might be associated with MASLD and early CVD. Imaging-based analyses of these associations have not been studied extensively in children. **Objectives**: To assess the reproducibility of liver and pancreatic steatosis and volume measurement on MRI in adolescents with obesity and MASLD and their association with homeostatic model assessment of insulin resistance (HOMA-IR) and subclinical vascular changes on ultrasound. **Methods**: This is an observational study on adolescents with MASLD and obesity. Hepatic and pancreatic steatosis, volume, and abdominal fat were assessed using magnetic resonance spectroscopy and proton density fat fraction. Reproducibility of these measurements was performed. Vascular markers included non-invasive vascular elastography (NIVE), carotid artery intima-media thickness (IMT), and pericardial fat thickness. Fasting blood tests measured the HOMA-IR. Bivariate correlation and simple linear regression were performed using SPSS. **Results**: We obtained 23 participants aged 12 to 17 years (78.3% male). Measurements were reproducible [ICC 0.807–0.998]. Liver steatosis was positively correlated with HOMA-IR (*p* = 0.015). Pancreas steatosis was positively correlated with HOMA-IR (*p* = 0.02), IMT/diameter (*p* = 0.002), and pericardial fat (*p* = 0.03). Liver steatosis was not significantly correlated with pancreas steatosis nor vascular markers. There were negative associations between NIVE metrics and visceral abdominal fat (*p* = 0.009) and intraperitoneal fat (*p* = 0.047). **Conclusions**: Liver and pancreas steatosis measurements on MRI are reproducible. In this exploratory study, adolescents with obesity and MASLD, pancreas steatosis, and pancreas volume show association with subclinical CVD markers. Visceral and intraperitoneal abdominal fat show association with increased vascular stiffness, suggesting a potential role of imaging-based cardiovascular risk assessment in this population if validated. These preliminary findings require validation in larger, diverse prospective cohorts.

## 1. Introduction

Metabolic dysfunction-associated steatotic liver disease (MASLD), previously non-alcoholic fatty liver disease (NAFLD), is on the rise among children and can lead to significant morbidity in adulthood [1]. Obesity, particularly abdominal fat, is a major risk factor for pediatric MASLD, which may progress to non-alcoholic steatohepatitis (NASH) and fibrosis [2]. MASLD is linked to cardiovascular complications, including high blood pressure and dyslipidemia, which increase the risk of atherosclerosis, coronary artery disease, and stroke in adulthood [3]. MASLD prevalence is higher in males than females, with evidence that girls may exhibit a relative protection before puberty that diminishes with hormonal changes later in life [2,4]. These differences are likely driven by interactions between sex hormones, genetics, and metabolic factors, and may also extend to cardiovascular risk markers. Early diagnosis could prevent early disease progression in children [5]. Despite this, few studies have focused on the imaging of pediatric obesity, MASLD, and cardiovascular health. Magnetic resonance imaging proton density fat fraction (MRI-PDFF) has shown strong diagnostic accuracy for hepatic steatosis [6,7].

Intima-media thickness (IMT) measurement evaluates arterial wall thickness and is valuable for early atherosclerosis detection, a risk factor for myocardial infarction and cerebrovascular strokes in adults [8,9]. Adolescent obesity correlates with greater arterial stiffness, though confounding factors warrant further study [10,11]. Non-invasive vascular elastography (NIVE) evaluates vessel wall biomechanics during cardiac pulsations and is a surrogate marker of vascular health in adolescents with obesity [11,12,13].

Pericardial fat, with its inflammatory role via cytokine release, is linked to arterial stiffness and endothelial dysfunction, both key factors in cardiovascular disease (CVD) development [14,15,16]. Increased pericardial fat thickness is linked to disrupted left ventricular energy metabolism, reduced coronary flow, and elevated CVD risk in adults [14,15,16]. Thus, assessing pericardial fat alongside IMT and vascular elastography markers could provide valuable insights into cardiovascular risk [17,18,19]. Imaging-based studies in pediatric patients assessing these associations are very scarce.

Pancreas steatosis, particularly in non-alcoholic fatty pancreas disease (NAFPD), shows a strong association with obesity and metabolic syndrome [20,21]. Elevated oxidative stress and systemic inflammation worsen both pancreatic and hepatic disease [22]. NAFPD is associated with impaired glucose metabolism, potentially exacerbating type 2 diabetes [22,23]. Additionally, there is emerging evidence of the association between pancreas steatosis and CVD markers in adults [22,24].

Metabolic syndrome, characterized by obesity, dyslipidemia, hypertension, and insulin resistance, is closely associated with abdominal fat distribution. Visceral adipose tissue (VAT) plays a key role in metabolic syndrome by acting both as fat storage and an endocrine tissue releasing adipokines, which disrupt glucose and lipid metabolism [25]. There is an association between VAT and early CVD markers [26,27]. While most studies consider intraperitoneal and retroperitoneal fat together as visceral fat, few reports have examined retroperitoneal fat’s independent role [28,29].

The objectives of this study are as follows: (1) To assess the reproducibility of MRI measures of hepatic and pancreatic steatosis and pericardial fat thickness. (2) To explore the association between liver and pancreas steatosis and subclinical vascular markers on imaging (IMT, NIVE, pericardial fat thickness), abdominal fat surface areas, and metabolic syndrome markers.

Quantitative assessment of liver and pancreas steatosis and its association with cardiovascular health is important in children to support a multidisciplinary approach aimed at halting disease progression.

## 2. Materials and Methods

### 2.1. Description

The study protocol received approval from the hospital’s ethics board, with assent and consent obtained from participants and their guardians. Conducted at an academic pediatric hospital, this is a sub-analysis of data obtained from a prospective open-label randomized clinical trial on the effect of polyphenol supplementation on liver steatosis in adolescents with MASLD and obesity at the initial visit (ClinicalTrials.gov ID: NCT03994029). The main study included 3 visits, in which the anthropometric measures and imaging were repeated [30].

### 2.2. Protocol

The complete research protocol was published in an open-access journal [30]. Eligibility criteria included age 12 to 17 years, Body Mass Index (BMI) > 85th percentile, and ongoing management for MALSD with standard clinical interventions. Exclusion criteria included recent significant weight change (>5% in the last 3 months), liver fat <5.5% on MR spectroscopy (MRS), diabetes, cystic fibrosis, pancreatitis, and other chronic illnesses [30]. Patients with diabetes were excluded, as the purpose is to investigate early-stage associations.

Various measurements were obtained: anthropometric data (weight in kg, height in cm, BMI in kg/cm^2^ using the formula weight/height^2^, and waist circumference in cm), blood pressure in mmHg, Tanner stage, previous medical history, medication intake, and 12-h fasting blood draws including glucose and insulin levels, which allow measuring of the homeostatic model assessment of insulin resistance (HOMA-IR) performed by a research assistant with more than 10 years of experience. HOMA-IR is calculated with the following formula: fasting glucose (mmol/L) × fasting insulin (mU/L)/22.5 [31]. Participants’ origin was noted. Abdominal and vascular imaging were obtained, and liver biopsy results (from the prior year, if available in the patient’s hospital chart) were used for correlation with imaging findings.

### 2.3. Image Acquisition

MRI scans were performed after a 12-h fast using a 1.5 T Ingenia MRI system (Philips Healthcare, Best, The Netherlands) with an abdominal coil, with participants in a supine position and aided by a belt for free respiration. Sequence parameters are shown in Table 1. The anterior antenna has 16 elements (dS Anterior). It is used with the posterior antenna, which has 12 elements (dS Posterior), for a total of 28 coil elements.

Feasibility was assessed by calculating the percentage of successful and interpretable exams for quantitative analysis of steatosis and volume. For reproducibility measurements, the observers were two medical students trained by a pediatric radiologist with 15 years of experience. The observers were blinded to each other and independently measured liver steatosis and volume, pancreas steatosis and volume, as well as pericardial fat thickness on the same MR exams. For intra-observer reproducibility, observer 1 repeated the same measurements after a one-month interval. The inter- and intra-operator reliabilities of the ultrasound subclinical vascular markers were assessed in a previous publication [13].

#### 2.3.1. Hepatic and Pancreatic Measurements

MR Spectroscopy

Liver steatosis was quantified using MRS, sampling the right hepatic lobe across three axial slices, avoiding the liver capsule and hepatic vessels, without water suppression (spectral width = 1250 Hz and spectral resolution = 1.22 Hz). We used jMRUI version 3.0 to analyze the SDAT file containing the acquired signal data. Echo times of 12, 18, 24, and 30 ms were used. Using a water spike at 4.7 ppm as a reference, we quantified lipid spikes. Residue verification was performed for quality check, followed by executing a Matlab (The Mathworks, version 2022b, Boston, MA, USA) script to calculate liver steatosis.

MRI-PDFF

MRI-PDFF was performed using the mDixonQuant sequence (Philips Intellispace Portal 12) with six echoes (first echo TE = 1.22 msec, echo spacing = 0.9 msec). For technical reasons we could not include the entire liver surface area; therefore, four elliptical regions of interest (ROI) were drawn manually on four different slices in the right hepatic lobe and pancreatic parenchyma (head, body, and tail) by two independent operators, and average values were used for statistical analysis. ROI area was 300 mm^2^ for liver measurements, avoiding liver capsule and vessel, and 50 mm^2^ for pancreas measurements, avoiding peri-pancreatic fat. Axial slices were selected by each observer at the largest section of each pancreatic region for ROI placement.

Liver and pancreas volumes

**Figure 1:**
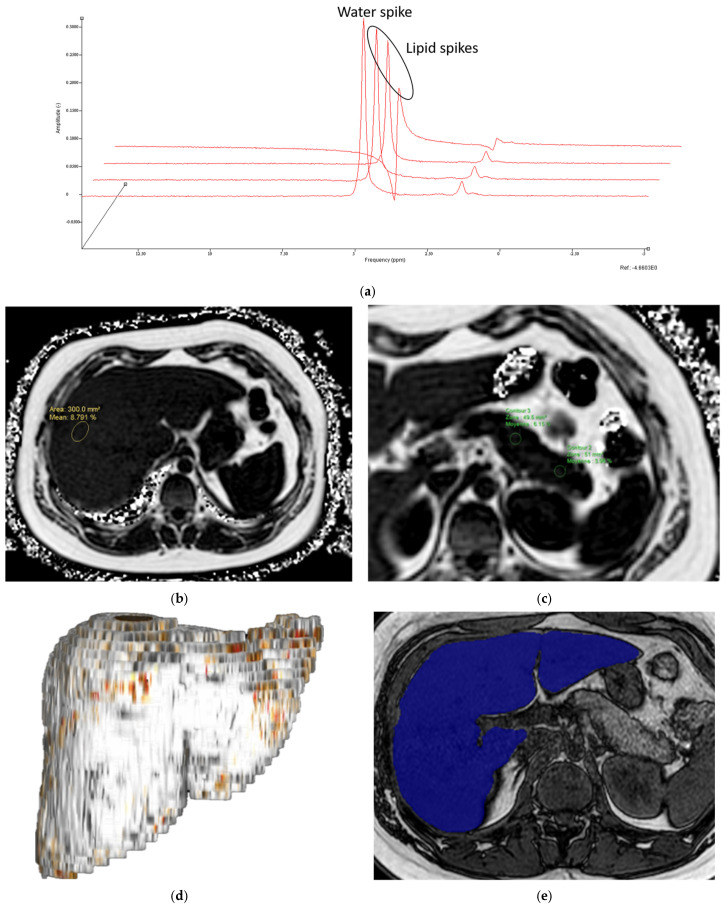
A 15-year-old female with (**a**) 8.69% liver steatosis measured by MRS and (**b**) 8.79% liver steatosis measured by MRI-PDFF using the mDixonQuant sequence. This same participant shows (**c**) pancreas steatosis of 3.98% in the tail and 6.15% in the body. (**d**) Volume reconstruction of the liver. (**e**) A single axial slice of the post-processed image used to calculate a liver volume of 2326.2 cm^3^ for the same participant. The same post-processing method generated pancreatic volume.

Volumes were measured on Philips Intellispace Portal 12 using semi-automatic delineation on axial slices of 6 mm thickness on the dual FFE-BH sequence for its better anatomic delimitation. Using the smart segmentation tool and slab mode, we delineated the entire liver or pancreas as ROI on all available slices, subtracting any erroneously selected perihepatic or peripancreatic fat when necessary. The automatic calculator function generated volume measurements of the selected ROIs (Figure 1).

#### 2.3.2. Subclinical Vascular Markers on Imaging

Carotid ultrasound

In line with Mannheim recommendations [32], participants were positioned supine with a 45° head tilt. Using a Terason uSmart3300 ultrasound system (Boston, MA, USA) with a 15 MHz linear array probe (15L4A, S/N: 20696), semi-automated measurement of the IMT was acquired on B-mode. Parameters were the following: depth of 3 or 4 cm, single focal zone, frame rate between 37 and 49 per second, dynamic range of 64, and focal range of 3. Using the integrated measurement tool in the uSmart3300 ultrasound system, operators generated the IMT values, aiming for a quality index > 0.5, and with less than 0.5 mm of variation between maximum and minimum IMT values. Measurements were performed on the posterior wall of the common carotid artery, along with the intima-to-intima diameter, allowing calculation of the IMT/diameter ratio.

Non-Invasive Vascular Elastography (NIVE)

The NIVE measurement was obtained using radiofrequency-based videos spanning five cardiac cycles. The video sequence of the common carotid artery was recorded 2 cm caudal to the carotid bulb using the same ultrasound system, with a sampling frequency of 30 MHz. Manual segmentation of the arterial wall was performed using C++ and Matlab (The Mathworks, version 2022b, Boston, MA, USA). The traced contour was then tracked and propagated on all remaining frames of the video sequence using an automatic segmentation method (analyzed at the Montreal University Hospital Research Center, Object Research Systems (ORS) Visual, Inc. Montreal, QC, Canada). Elastography measured axial and lateral translation as well as strain, with “axial” and “lateral” referring to directions parallel and perpendicular to the ultrasound beam, respectively. The software generated consequently cumulated axial strain (CAS in %) and cumulated axial translation (CAT in mm) metrics over the entire frames. The CAS/CAT ratio was then calculated.

Measurement of pericardial fat thickness

**Figure 2:**
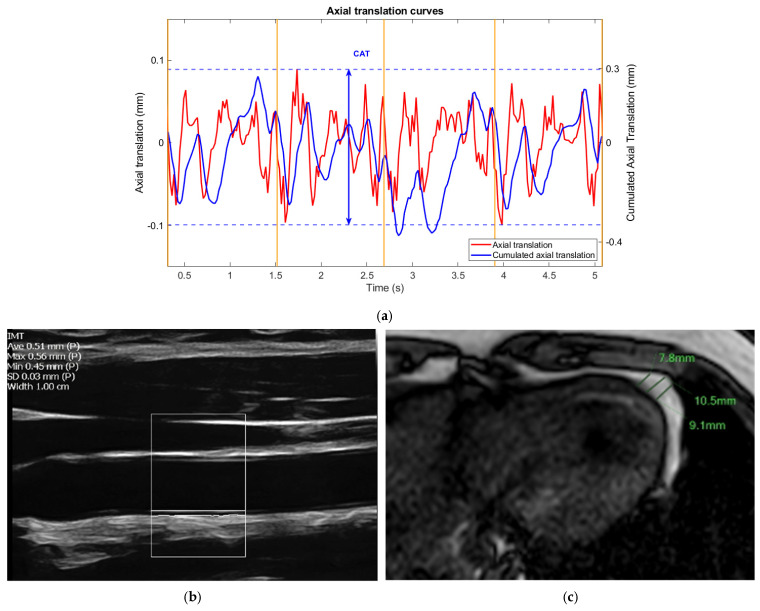
Early vascular markers on a 17-year-old male participant. (**a**) Cumulated axial translation measurement of NIVE metrics on a carotid ultrasound. Each yellow line represents one cardiac cycle, with the *x*-axis indicating time in seconds. The blue tracing represents cumulated axial translation (in mm) on the *y*-axis. (**b**) IMT measurement of the common carotid artery on a longitudinal ultrasound image, showing the automated calculation of IMT (0.51 mm) over a 1 cm segment of the vessel wall (the width of the white box). The standard deviation is 0.03 mm, indicating the robustness of the measurement. (**c**) Peri-apical fat measurements on a single axial MRI slice of the same participant, shown in green lines.

Following existing methods [33,34], pericardial fat thickness was measured as the average thickness of peri-coronary and peri-apical fat. Peri-coronary fat refers to the adipose tissue between the heart’s surface and the pericardium surrounding the main coronary arteries. Peri-apical fat is the adipose tissue located between the apex of the heart and the pericardium. Measurements were taken from single axial images of the dual FFE-BH sequence, with three readings each for peri-coronary and peri-apical fat, then averaged for analysis (Figure 2).

#### 2.3.3. Measurement of Abdominal Fat Compartment Surface Area and Thickness

**Figure 3:**
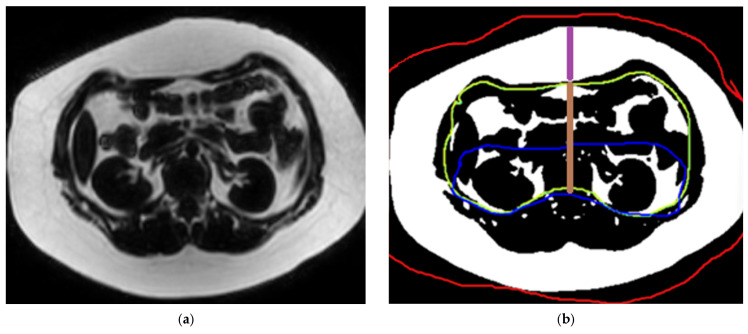
A 17-year-old male participant with BMI = 41 kg/m^2^. (**a**) A single axial slice at the L2 level was processed on the ImageJ software. (**b**) The resulting image shows the fat area segmentation. The red ROI encompasses the entire abdomen, representing “Total fat area”. The green ROI highlights abdominal fat labelled “VAT area”, and the blue ROI isolates retroperitoneal fat. The purple line shows SAT thickness, while the brown line indicates VAT thickness.

The abdominal fat compartment measurement method was based on Trout et al. [35]. Fat areas were measured on the MRI-PDFF sequence using a single axial image at the midportion of the L2 vertebral body on ImageJ software (v1.53t 2022). The image was processed to isolate fat regions by setting a threshold, and then desired ROIs were delineated (Figure 3). The measurements (in mm^2^) included total fat area: combined fat within the abdominal wall and cavity; visceral fat (VAT) area: fat enclosed by abdominal wall muscles; retroperitoneal fat area: abdominal fat bound anteriorly by a line intersecting the ascending and descending colon; intraperitoneal fat area: VAT minus retroperitoneal fat area; subcutaneous fat area (SAT): total fat area minus VAT; midline VAT thickness: distance in mm from the inner rectus muscle to the anterior edge of the L2 vertebral body; and midline SAT thickness: distance in mm from the skin surface to the inner rectus muscle.

#### 2.3.4. Liver Elastography

**Figure 4:**
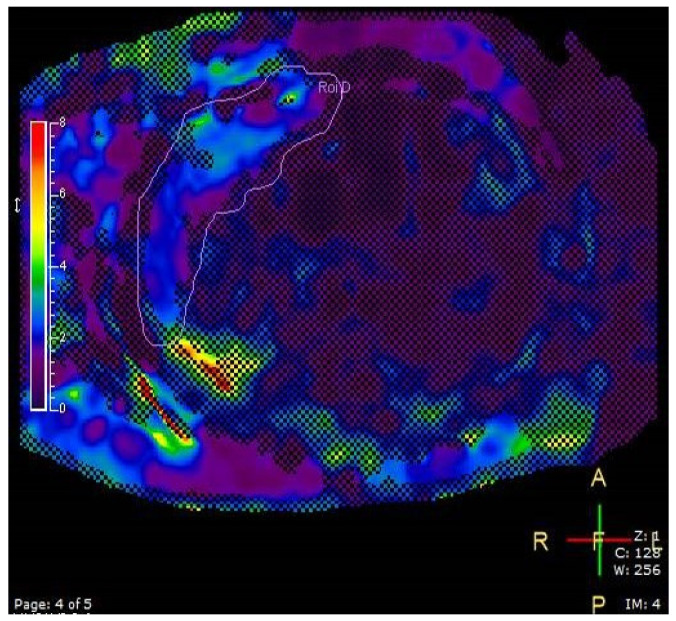
A single ROI on one slice of MR elastography in a 17-year-old male participant with hepatic elasticity of 2.03 ± 0.65 kPa and BMI = 41 kg/m^2^. Cross-shaded areas represent poor wave propagation and are automatically excluded from the ROI by the software.

MR elastography was used to assess liver stiffness [36]. A Resoundant system with a pneumatic oscillator set to an amplitude of 60 Hz was connected to a Philips 1.5T Ingenia MRI system (Philips Healthcare, Best, The Netherlands) to acquire 2D GRE images. Four liver ROIs are automatically measured across four different slices that are evenly distributed from cranial to caudal regions of the liver (Figure 4).

### 2.4. Statistics

Statistical analyses included descriptive evaluations of anthropometric and laboratory variables. Data normality was tested using Shapiro–Wilk. Inter- and intra-observer reproducibility was assessed with the intraclass correlation coefficient (ICC) for liver, pancreas, and pericardial fat measurements. ICC (2,1) was used for interrater reproducibility, with values between 0.75 and 0.9 indicating good reliability, and values greater than 0.9 indicating excellent reliability [37]. Using data collected by observer 1, comparative analyses were conducted between liver imaging modalities (MRS, mDixon-Quant), pancreas imaging, and subclinical vascular markers using Spearman’s coefficient, as well as simple linear regression analyses when appropriate. Similarly, associations between the abdominal fat compartment, subclinical vascular markers, and HOMA-IR were analyzed using Spearman. For categorical variables, Mann–Whitney and Kruskall–Wallis tests were used. In this exploratory observational study, correction for multiple correlations is not applicable, according to the biostatistician. All analyses were conducted on IBM SPSS Statistics (Version 29) under biostatistician guidance (TP and MS).

## 3. Results

### 3.1. Anthropometric

**Figure 5:**
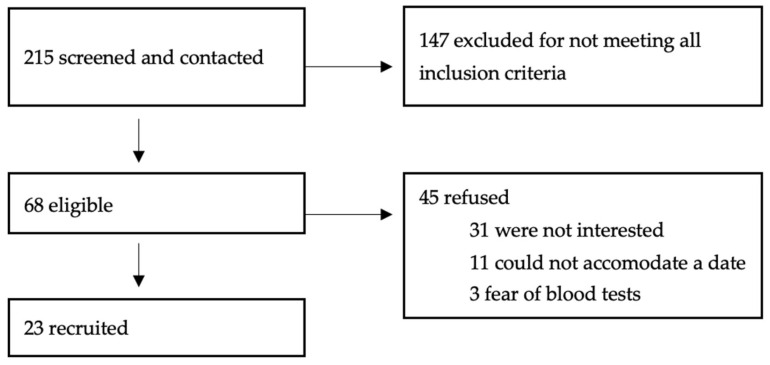
Flowchart of recruitment at the initial visit. A total of 23 participants were obtained for the correlation analyses and 58 MRIs (across all three visits) for the purpose of reproducibility analyses.

The study included 23 participants (Figure 5): 18 males (78.3%), aged 12–17 years (mean age 14.8 ± 1.7 years) (Table 2). Participants’ origin was noted; however, the sample size for each subgroup was too small to perform stratification analysis.

### 3.2. Correlation Between Imaging Modalities and Reproducibility

All available imaging was of good quality and analyzable. Only 6 out of 23 participants (26%) had undergone liver biopsies. The correlation between liver biopsy results and MRI-PDFF or MRS did not reach statistical significance (see Tables S1 and S2 Supplemental Material). Based on Spearman’s bivariate analysis, liver steatosis measured on MRI-PDFF and MRS were positively and strongly correlated with each other (ρ = 0.84; 95% confidence interval (CI) [0.63–0.94; *p* < 0.001]). Measurement reproducibility was high for liver and pancreas measurements, as well as for pericardial fat measurements, with ICC values ranging from good to excellent for both intra- and inter-operator correlation (Table 3). Since measurements were reproducible, we used data from the first observer for subsequent analyses.

### 3.3. Liver Steatosis

There were no significant correlations between age, sex, height, weight, BMI, and MRI-PDFF or subclinical vascular markers (see Table S3 Supplementary Material). We did not find significant correlation using Spearman’s ρ between liver steatosis and the subclinical vascular markers in the data collected (Table 4). We then performed simple linear regressions between liver steatosis, IMT, and NIVE markers. We observed several trends, though none were statistically significant. These include the association between MRS and CAS/CAT (r^2^ = 0.15, *p* = 0.08) and between MRI-PDFF and IMT/diameter (r^2^ = 0.12, *p* = 0.16).

A moderate to strong positive correlation was found between liver steatosis (on both MRS and MRI-PDFF) and HOMA-IR.

### 3.4. Elastography

On MR elastography, only 9% of participants had abnormally elevated liver stiffness exceeding 2.77 kPa [39,40]. Liver elastography was positively correlated with liver volume (ρ = 0.52; 95% CI [0.099–0.78]; *p* = 0.016) but did not show significant correlation with liver steatosis (*p* = 0.56).

### 3.5. Pancreas MRI-PDFF

Most pancreas measurements, including volume and steatosis, showed significant positive correlations with weight, waist circumference, and BMI (see Table S3 Supplementary Material). Pancreatic head steatosis also had a significant correlation with age (ρ = 0.56 [0.15–0.80]) and Tanner stage, but no other pancreas measurements showed an association with age, sex, Tanner stage, origin, or height (see Table S3 and S4 Supplementary Material).

Pancreas volume showed a strong significant positive correlation with pancreas steatosis (ρ = 0.68 [0.33–0.86]) and a moderate significant correlation with liver volume (ρ = 0.44 [0.00–0.74]) and HOMA-IR (ρ = 0.65 [0.25–0.86]).

As shown in Table 4, pancreas steatosis was positively correlated with peri-apical fat, and pancreas volume was positively correlated with pericardial fat thickness. To further explore these associations, simple linear regression analyses were performed, which demonstrated a positive and statistically significant correlation between the pancreas volume and the pericardial fat thickness (r^2^ = 0.29, *p* = 0.012) and between the pancreas volume and IMT/diameter (r^2^ = 0.37, *p* = 0.009). Additionally, pancreas steatosis was positively correlated with pericardial fat thickness (r^2^ = 0.24, *p* = 0.025) and with IMT/diameter (r^2^ = 0.45, *p* = 0.003).

### 3.6. Measurement of Abdominal Fat Surface Area and Thickness

Most abdominal fat measurements showed significant positive correlations with weight, waist circumference, and BMI, but no associations with age, sex, height, or origin (see Table S3 and S4 Supplementary Material).

Moderate to strong positive associations were observed between peri-apical fat and all abdominal fat compartments, except subcutaneous fat, which correlated moderately with pericardial fat thickness. We obtained moderate negative associations between VAT and CAT, as well as between intraperitoneal fat and both CAT and CAS. HOMA-IR was moderately to strongly positively correlated with VAT, intraperitoneal, and total fat areas (Table 4).

Finally, both pancreas volume and steatosis showed moderate to strong significant positive Spearman correlations with abdominal fat compartments, namely, VAT (ρ = 0.53 [0.095–0.79] *p* = 0.02; ρ = 0.61 [0.22–0.83] *p* = 0.004), intraperitoneal fat (ρ = 0.54 [0.11–0.80] *p* = 0.01; ρ = 0.70 [0.35–0.87] *p* < 0.001), and total fat areas (ρ = 0.59 [0.18–0.82] *p* = 0.007; ρ = 0.57 [0.15–0.81] *p* = 0.009). Only pancreas volume was correlated with subcutaneous fat (ρ = 0.51 [0.069–0.78] *p* = 0.02).

## 4. Discussion

### 4.1. Reproducibility of Imaging Measurements

The MRI-PDFF sequence is feasible and reproducible in children with obesity and MASLD, which is in line with findings from adult and pediatric literature [6,7]. We also observed a strong correlation between MRS and MRI-PDFF measurements. However, liver imaging did not show a statistically significant correlation with liver biopsy results, likely due to the small number of participants who underwent a biopsy.

### 4.2. Exploring Associations Between Subclinical Vascular Markers and MASLD

We did not find an association between liver MRI-PDFF and the IMT/diameter, nor between liver steatosis and CAS/CAT. It is, however, known that liver steatosis may exacerbate atherosclerosis by disrupting lipoprotein metabolism and increasing oxidative stress, leading to vascular wall fat deposition and altered vascular wall mechanics [3].

Most pediatric studies assessed liver steatosis using ultrasound [3]. Most demonstrated an increase in IMT when comparing children with high liver steatosis to controls. Two studies further showed that IMT was increased in groups with more severe liver steatosis as measured by ultrasound [41,42]. However, another study, which showed an increase of IMT and epicardial fat in a group with liver steatosis on ultrasound compared to those without, did not find a significant correlation between IMT, epicardial fat, and increasing steatosis grade [43].

Studies using MRI to assess liver steatosis show conflicting results. Using a 5% cutoff for liver steatosis on MRS, Koot et al. did not find significant changes in IMT or arterial wall stiffness in children with steatosis and obesity [44]. A different study evaluating a population of 10-year-old children reported that liver steatosis was inversely associated with IMT and distensibility [45]. The findings may be explained by the inclusion of many healthy, non-obese children. In such conditions, IMT may reflect physiological remodeling of the media layer in response to somatic growth rather than pathological changes. This could also apply to our cohort with respect to the differences in Tanner stage and physiologic change.

Regarding NIVE metrics, previous findings from a longitudinal cohort study on childhood obesity show that obesity may be associated with a higher CAT and lower CAS/CAT ratio; therefore, a lower axial strain per unit of axial translation would indicate arterial stiffness in the elevated BMI group [11]. However, a prospective study on HIV patients determined that reduced carotid wall translation can also indicate a stiffer carotid artery wall, exerting less mobility [12]. We hypothesize that higher deformation by the translation unit of the vessel wall could be an early compensatory phase for vascular wall mechanical modifications. The duration of obesity and liver steatosis could play a role in the onset of vascular change, but this could not be assessed in the present cohort.

### 4.3. Exploring Associations Between Subclinical Vascular Markers and Pancreas Measurements

Our preliminary findings show that pancreas volume and steatosis are positively correlated with pericardial fat thickness and IMT/diameter. This aligns with findings in adult populations supporting the link between pancreas steatosis and subclinical vascular changes [22,24]. We are not aware of pediatric radiology studies on the association between pancreatic measurements and subclinical vascular markers, nor of validated imaging cut-off values for fatty pancreatic disease in children.

Furthermore, pancreas volume and steatosis correlated positively with VAT, intraperitoneal fat, and total fat areas, as well as with weight, waist circumference, and BMI. This is consistent with Trout et al., who reported that pancreatic steatosis was positively associated with weight, BMI, absolute abdominal fat area, and midline abdominal fat thickness [35]. In another pediatric study, pancreatic steatosis was significantly correlated with VAT, and the initial association between pancreatic steatosis and hepatic steatosis lost significance after adjusting for age, gender, Tanner stage, BMI, and VAT [46].

Further research, including prospective longitudinal studies, is needed to validate the role of pancreas steatosis and volume as imaging markers. These could help guide and explore interventions aimed at mitigating future cardiovascular risks in MASLD patient management.

### 4.4. Exploring Associations Between Subclinical Vascular Markers and Abdominal Fat Compartments

Our preliminary findings show strong correlations between peri-apical fat thickness and abdominal fat compartments, particularly VAT and intraperitoneal fat. Subcutaneous fat tissue thickness also moderately correlated with pericardial fat thickness. Lower CAT was associated with higher VAT and intraperitoneal fat, indicating lower vascular translation during systole and suggesting early changes of decreased vascular elasticity. Negative correlations between intraperitoneal fat and CAS indicate less vascular wall deformation and therefore increased vascular stiffness. These findings are in line with the existing literature that supports the link between obesity, abdominal fat stores, and subclinical vascular changes [11,25,27]. This suggests a potential role for monitoring abdominal fat in children with obesity.

### 4.5. Insulin Resistance

Our preliminary findings show significant positive correlations between HOMA-IR and liver steatosis, pancreas volume, pancreas steatosis, total abdominal fat, VAT, and intraperitoneal fat. These results are consistent with the existing literature [2,22,25]. Interestingly, retroperitoneal fat did not correlate with HOMA-IR. This suggests that retroperitoneal and intraperitoneal fat may have distinct biological roles, with intraperitoneal fat being more likely associated with metabolic syndrome risk due to its drainage into the portal vein, which emphasizes the link between insulin resistance and specific abdominal fat distributions [28,29]. This suggests the potential need for comprehensive metabolic and cardiovascular evaluations in children with MASLD [3].

### 4.6. Limitations

This cross-sectional study had a small sample size (*n* = 23), limiting statistical power and the ability to detect significant associations. High reproducibility of measurements supported the robustness of our approach using repeated MRI and vascular ultrasound measures. All imaging was performed on a Philips MRI 1.5 T machine, which may not be generalizable to other vendors. MRI-PDFF liver steatosis did not include the entire liver surface, as four small ROIs were used, which could be a potential sampling bias. In this exploratory study, results should be interpreted with caution due to the large number of models analyzed and the presence of large confidence intervals. In our cohort, the limited number of female participants did not allow for a reliable sex-stratified statistical analysis. This prevented us from fully addressing gender-specific patterns of disease presentation and vascular markers. Future research should include larger, sex-balanced, and ethnically diverse cohorts and examine the effects of the duration of obesity on vascular changes. The number of available biopsies was insufficient to assess correlations; however, PDFF and MR are considered the gold standard imaging tools and are well validated. Finally, associations were not evaluated in children with normal weight.

## 5. Conclusions

This cross-sectional exploratory study demonstrates the good reproducibility of quantitative MRI measurements of liver steatosis and pancreas steatosis in adolescents with obesity. In children with obesity, we found positive associations between pancreas steatosis, pancreas volume, abdominal fat compartment, and subclinical vascular markers on imaging. Additionally, imaging-based liver steatosis, pancreas steatosis, and abdominal fat compartments were associated with HOMA-IR, a marker of insulin resistance. These preliminary findings warrant validation in larger, diverse prospective cohorts with respect to origin and sex. If validated, a multidisciplinary approach, including radiologists, may be crucial for diagnosis, follow-up, and early intervention to mitigate long-term risk.

## Figures and Tables

**Table 1:** Sequence parameters of all MR sequences acquired and used on a 1.5 T MR magnet.

	MR Elastography	PDFF	MRS	FEE-BH Dual
Sequence	MR Elastography-4SL, Fast field echo (FFE)	mDixon- Quant-BH FFE	Steam-mTE Echo	Ax dual-FFE-BH
FOV (mm)	400	400	-	340
Matrix	272 × 64	144 × 116	-	208 × 128
Number of slices	4	70	-	28
Slice thickness (mm)	10	6	Spectroscopic volume of interest 25 mm^3^	6
TE (msec)	20	1.19	12	2.3
TR (msec)	50	6.7	3000	105
Slice orientation	Transverse	Transverse	Transverse	Transverse
Gap (mm)	1	0	-	1.2
Flip angle (degree)	20	5	90	75
Time (s)	5 × 13	1 × 14	14 (3 sequences)	2 × 7
Parallel imaging	2 (SENSE)	-	-	2 (SENSE)
Breath hold	Yes (after expiration)	Yes	No	Yes

**Table 2:** Descriptive and anthropometric characteristics of the entire cohort of participants (*N* = 23) at baseline.

Variables	Mean ± Standard Deviation
Sex, No. (%)	
Males	18 (78.2%)
Females	5 (21.8%)
Age (years)	14.8 ± 1.7
Origin, No. (%)	
Caucasian	12 (52.2%)
Hispanic	5 (21.8%)
First Nation	3 (13%)
Other	3 (13%)
Tanner Stage, No. (%)	
1	1 (4.4%)
2	2 (8.7%)
3	7 (30.4%)
4	7(30.4%)
5	6 (26.1%)
Weight (kg)	108.0 ± 24.3
Weight percentile	96.1 ± 2.4
Height (cm)	173.8 ± 8.0
Height percentile	79.8 ± 21.9
BMI (kg/m^2^)	35.5 ± 6.7
BMI percentile	98.6 ± 2.3
Liver steatosis PDFF	20.84 ± 11.4
Systolic blood pressure (mm Hg)	124 ± 20
Waist circumference (cm)	112.4 ± 17.9
Glucose * (mmol/L)	5.2 ± 0.5
Triglycerides * (mmol/L)	1.5 ± 0.7
Insulin * (pmol/L)	195.0 ± 98.8

* The normal range in our pediatric hospital for glucose is 3.9–5.8 mmol/L, for insulin 15.2–345.0 pmol/L, and for triglycerides 0.40–1.30 mmol/L [38].

**Table 3:** Intraclass correlation coefficient for inter- and intra-operator correlation.

	ICC Inter-Operator	95% CI	ICC Intra-Operator	95% CI
**Liver volume**	0.996	0.984–0.999	0.988	0.949–0.997
**Liver PDFF**	0.998	0.992–0.999	0.995	0.982–0.998
**Pancreas volume**	0.995	0.982–0.999	0.995	0.981–0.999
**Pancreas PDFF (average)**	0.987	0.957–0.996	0.971	0.902–0.992
**Pancreas PDFF (tail)**	0.970	0.898–0.991	0.934	0.793–0.980
**Pancreas PDFF (body)**	0.984	0.945–0.995	0.961	0.873–0.988
**Pancreas PDFF (head)**	0.953	0.845–0.986	0.926	0.763–0.978
**PFT**	0.970	0.884–0.992	0.985	0.943–0.996
**Peri-coronary fat**	0.865	0.549–0.965	0.878	0.588–0.968
**Peri-apical fat**	0.961	0.852–0.990	0.807	0.383–0.949

PFT = pericardial fat thickness, which is the average measurement of peri-coronary fat and peri-apical fat.

**Table 4:** Bivariate relationships (Spearman’s ρ) between subclinical vascular markers, serum insulin resistance, liver, pancreas, and abdominal fat measurements at visit 1 for all subjects (*N* = 23).

		IMT	IMT/ Diameter	CAT	CAS	CAS/CAT	PFT	Peri Coronary Fat	Peri Apical Fat	HOMA-IR
*Liver* *volume*	ρ [95% CI]	0.024 [−0.423–0.462]	−0.038 [−0.473–0.412]	0.20 [−0.278–0.600]	−0.20 [−0.598–0.281]	−0.33 [−0.684–0.141]	0.19 [−0.273–0.586]	0.17 [−0.291–0.572]	0.28 [−0.187–0.643]	0.28 [−0.225–0.671]
	*p*-value	0.92	0.87	0.39	0.40	0.15	0.40	0.45	0.22	0.25
*Liver PDFF*	ρ [95% CI]	−0.012 [−0.453–0.433]	0.039 [−0.411–0.474]	−0.29 [−0.656–0.190]	−0.051 [−0.493–0.412]	0.27 [−0.213–0.642]	−0.20 [−0.591–0.265]	−0.16 [−0.566–0.301]	−0.20 [−0.590–0.268]	0.55 * [0.115–0.809]
	*p*-value	0.96	0.87	0.22	0.83	0.26	0.38	0.48	0.39	0.015
*MRS*	ρ [95% CI]	0.033 [−0.405–0.459]	0.025 [−0.412–0.453]	−0.35 [−0.687–0.109]	−0.075 [−0.501–0.380]	0.32 [−0.144–0.668]	0.036 [−0.413–0.472]	−0.042 [−0.476–0.409]	−0.064 [−0.492–0.390]	0.66 * [0.283–0.862]
	*p*-value	0.88	0.91	0.12	0.75	0.16	0.88	0.86	0.78	0.002
*Pancreas* *volume*	ρ [95% CI]	0.085 [−0.371–0.509]	0.057 [−0.396–0.488]	−0.13 [−0.553–0.342]	−0.38 [−0.711–0.090]	−0.12 [−0.544–0.353]	0.49 * [0.055–0.764]	0.33 [−0.131–0.675]	0.49 * [0.063–0.768]	0.65 * [0.251–0.861]
	*p*-value	0.71	0.81	0.58	0.099	0.61	0.026	0.14	0.023	0.003
*Pancreas PDFF* *(average)*	ρ [95% CI]	−0.18 [−0.577–0.286]	−0.013 [−0.453–0.432]	−0.10 [−0.530–0.370]	−0.23 [−0.619–0.250]	−0.081 [−0.516–0.387]	0.32 [−0.141–0.669]	−0.04 [−0.474–0.410]	0.67 * [0.325–0.859]	0.53 * [0.071–0.805]
	*p*-value	0.44	0.96	0.67	0.33	0.73	0.15	0.86	<0.001	0.023
*Pancreas PDFF (tail)*	ρ [95% CI]	−0.13 [−0.543–0.330]	−0.012 [−0.452–0.433]	−0.13 [−0.549–0.347]	−0.16 [−0.570–0.320]	−0.030 [−0.477–0.430]	0.46 * [0.015–0.747]	0.088 [−0.369–0.511]	0.73 * [0.429–0.887]	0.70 * [0.331–0.882]
	*p*-value	0.57	0.96	0.60	0.51	0.90	0.038	0.70	<0.001	0.001
*Pancreas PDFF (body)*	ρ [95% CI]	−0.23 [−0.611–0.236]	−0.11 [−0.525–0.352]	−0.17 [−0.576–0.312]	−0.34 [−0.688–0.135]	−0.071 [−0.508–0.396]	0.31 [−0.159–0.659]	0.015 [−0.431–0.455]	0.55 * [0.143–0.799]	0.62 * 0.202–0.847]
	*p*-value	0.31	0.64	0.49	0.14	0.77	0.18	0.95	0.010	0.006 [
*Pancreas PDFF (head)*	ρ [95% CI]	−0.089 [−0.512–0.368]	0.079 [−0.377–0.504]	−0.19 [−0.595–0.285]	−0.20 [−0.598–0.281]	0.023 [−0.436–0.471]	0.23 [−0.240–0.609]	−0.059 [−0.489–0.394]	0.64 * [0.280–0.845]	0.35 [−0.152–0.711]
	*p*-value	0.70	0.73	0.41	0.40	0.93	0.32	0.80	0.002	0.15
*Total fat area*	ρ [95% CI]	0.003 [−0.451–0.456]	−0.008 [−0.460–0.448]	−0.17 [−0.587–0.324]	−0.24 [−0.636–0.252]	−0.03 [−0.489–0.442]	0.43 [−0.026–0.741]	0.25 [−0.230–0.632]	0.45 * [−0.003–0.751]	0.57 * [0.102–0.828]
	*p*-value	0.99	0.98	0.50	0.32	0.90	0.056	0.29	0.046	0.018
*Visceral fat area (VAT)*	ρ [95% CI]	−0.099 [−0.529–0.372]	−0.12 [−0.541–0.357]	−0.58 * [−0.823–−0.155]	−0.37 [−0.714–0.113]	0.29 [−0.204–0.665]	0.26 [−0.223–0.637]	0.032 [−0.428–0.479]	0.57 * [0.155–0.813]	0.54 * [0.071–0.818]
	*p*-value	0.68	0.63	0.009	0.12	0.23	0.27	0.90	0.009	0.024
*Retroperitoneal fat area*	ρ [95% CI]	0.37 [−0.098–0.707]	0.35 [−0.128–0.691]	−0.32 [−0.681–0.176]	−0.17 [−0.586–0.326]	0.21 [−0.286–0.614]	0.36 [−0.108–0.702]	0.23 [−0.246–0.622]	0.53 * [0.099–0.793]	0.16 [−0.359–0.606]
	*p*-value	0.11	0.14	0.19	0.50	0.40	0.12	0.32	0.016	0.54
*Intraperitoneal fat area*	ρ [95% CI]	−0.26 [−0.637–0.223]	−0.26 [−0.636–0.224]	−0.57 * [−0.818–−0.140]	−0.46 * [−0.763–0.005]	0.20 [−0.291–0.610]	0.22 [−0.257–0.614]	−0.031 [−0.478–0.429]	0.53 * [0.102–0.793]	0.62 * [0.176–0.850]
	*p*-value	0.27	0.28	0.011	0.047	0.41	0.35	0.90	0.016	0.009
*Subcutaneous area (SAT)*	ρ [95% CI]	0.062 [−0.403–0.501]	0.029 [−0.431–0.476]	0.00 [−0.466–0.466]	−0.14 [−0.571–0.345]	−0.12 [−0.553–0.368]	0.46 * [0.006–0.755]	0.32 [−0.153–0.678]	0.34 [−0.133–0.688]	0.45 [−0.056–0.771]
	*p*-value	0.80	0.91	1.0	0.56	0.63	0.042	0.17	0.14	0.071
*Thickness VAT*	ρ [95% CI]	−0.12 [−0.544–0.353]	0.12 [−0.357–0.541]	−0.19 [−0.602–0.303]	−0.29 [−0.665–0.204]	−0.056 [−0.508–0.420]	0.22 [−0.259–0.613]	0.063 [−0.402–0.503]	0.36 [−0.108–0.701]	0.63 * [0.203–0.858]
	*p*-value	0.62	0.63	0.44	0.23	0.82	0.35	0.79	0.12	0.006
*Thickness SAT*	ρ [95% CI]	−0.018 [−0.468–0.439]	−0.079 [−0.514–0.389]	0.038 [−0.436–0.495]	−0.18 [−0.599–0.308]	−0.13 [−0.563–0.356]	0.096 [−0.374–0.526]	0.24 [−0.243–0.623]	0.009 [−0.447–0.461]	−0.017 [−0.505–0.479]
	*p*-value	0.94	0.74	0.88	0.45	0.59	0.69	0.32	0.97	0.95

IMT = intima-media thickness; CAS = cumulated axial strain; CAT = cumulated axial translation; PFT = pericardial fat thickness; IMT/diameter = IMT/intima-to-intima diameter; HOMA-IR = homeostatic model assessment of insulin resistance. * Statistically significant results (*p* < 0.05).

## Data Availability

Available upon reasonable request.

## Supplementary Materials

The following supporting information can be downloaded at: https://www.mdpi.com/article/10.3390/jcm14197048/s1. Table S1: Descriptive liver biopsy findings in *n* = 6 participants. Table S2: Kruskall Wallis and Mann Whitney U tests for comparison between liver biopsy findings and liver MR elastography, spectroscopy and PDFF (*n* = 6). Table S3: Spearman correlation between age, anthropometric measurements and all liver, pancreas, abdominal fat compratment, and cardiovascular measurements (*n* = 23). Table S4: Kruskall Wallis test for origin and Tanner stage, and Mann Whitney U test for sex, to compare with all liver, pancreas, abdominal fat compartment, and cardiovascular measurements (*n* = 23).

## Abbreviations

The following abbreviations are used in this manuscript:

MASLD	metabolic dysfunction-associated steatosic liver disease
NIVE	non-invasive vascular elastography
IMT	intima-media thickness
HOMA-IR	homeostatic model assessment of insulin resistance
MRI-PDFF	magnetic resonance imaging proton density fat fraction
VAT	visceral adipose tissue
