# Supplementary material for: Relationship Between Liver Steatosis, Pancreas Steatosis, Metabolic Comorbidities, and Subclinical Vascular Markers in Children with Obesity: An Imaging-Based Study
Source: J Clin Med. 2025 Oct 6;14(19):7048. doi: 10.3390/jcm14197048 (PMC12525386; doi:10.3390/jcm14197048)
Supplement: Supplementary file 1 [file jcm-14-07048-s001.zip › jcm-3854249-supplementary.pdf]

Relationship Between Liver Steatosis, Pancreas Steatosis, Metabolic Comorbidities, and Subclinical Vascular Markers in Children with Obesity: An Imaging-Based Study

Kenza El Ghomari [1,*], Anna Voia [1], Jean-Baptiste Moretti [1], Anik Cloutier [2], Guy Cloutier [3] and Ramy El Jalbout [1,2,*]

[1] Department of Radiology, Faculty of Medicine, Université de Montréal, Montreal, QC H3C3J7, Canada
[2] Centre de Recherche du Centre Hospitalier Universitaire Sainte-Justine (CRCHUSJ), Montreal, QC H3T1C5, Canada
[3] Centre de Recherche du Centre Hospitalier de L'Université de Montréal (CRCHUM), Montreal, QC H2X0A9, Canada
* Correspondence: kenza.el.ghomari@umontreal.ca (K.E.G.); ramy.el-jalbout.med@ssss.gouv.qc.ca (R.E.J.)

Supplemental Material

Table S1. Descriptive liver biopsy findings in N=6 participants.

Liver biopsy	N= 6 [% of participants]
**Steatosis grade**	
0	0 [0%]
1	1 [17%]
2	2 [33%]
3	3 [50%]
**Lobular inflammation**	
0	0 [0%]
1	3 [50%]
2	2 [33%]
3	1 [17%]
**Fibrosis**	
0	0 [0%]
1	3 [50%]
2	3 [50%]
3	0 [0%]
4	0 [0%]
**Hepatocyte ballooning**	
Present	2 [33%]

Table S2. Kruskall Wallis and Mann Whitney U tests for comparison between liver biopsy findings and liver MR elastography, spectroscopy and PDFF (N=6).

	**MR elastography**	**MR spectroscopy**	**Liver PDFF**
**Steatosis grade**	2.45	0.24	2.4

	p-value	0.29	0.89	0.30
**Lobular inflammation**		3.97	0.86	0.33
	p-value	0.14	0.65	0.56
**Fibrosis**		1.23	0.048	1.33
	p-value	0.27	0.83	0.25
**Hepatocyte ballooning**		U=0.00 Z= -1.88	U= 4.00 Z= 0.00	U= 0.00 Z= -1.4
	p-value	0.13	1.00	0.40

Table S3. Spearman correlation between age, anthropometric measurements and all liver, pancreas, abdominal fat compratment, and cardiovascular measurements (N=23).

	**Age**	**Height**	**Weight**	**BMI**	**Waist circumference**	**Systolic BP**	**Glucose**	**Triglycerides**	**TG index**
**Liver volume**	0.087	0.238	0.390	0.344	0.216	-0.112	-0.143	-0.456	-0.402
p-value [CI]	0.708 [-0.370 - 0.510]	0.298 [-0.228 - 0.616]	0.081 [-0.064 - 0.710]	0.126 [-0.116 - 0.683]	0.348 [-0.251 - 0.601]	0.629 [-0.528 0.348]	0.560 [-0.570 0.346]	0.038 [-0.748 -0.017]	0.088 [-0.731 0.079]
**Liver PDFF**	0.025	0.112	0.038	0.005	0.214	0.404	0.370	0.216	0.256
p-value [CI]	0.915 [-0.423 - 0.462]	0.630 [-0.348 - 0.528]	0.871 [-0.412 - 0.473]	0.984 [-0.439 - 0.446]	0.351 [-0.252 - 0.600]	0.069 [-0.047 0.718]	0.108 [-0.100 0.706]	0.348 [-0.251 0.601]	0.277 [-0.224 0.636]
**MR Spectroscopy**	-0.196	0.099	0.263	0.219	0.409	0.086	0.410	0.047	0.077
p-value [CI]	0.382 [-0.579 - 0.258]	0.662 [-0.349 - 0.510]	0.238 [-0.192 - 0.624]	0.328 [-0.236 - 0.595]	0.059 [-0.028 - 0.715]	0.704 [-0.360 0.500]	0.072 [-0.053 0.728]	0.836 [-0.394 0.470]	0.748 [-0.391 0.513]
**Pancreas volume**	0.132	0.221	0.626*	0.459*	0.587*	-0.239	-0.043	-0.344	-0.416
p-value [CI]	0.567 [-0.330 - 0.543]	0.336 [-0.246 - 0.604]	0.002 [0.253 – 0.837]	0.037 [0.020 – 0.749]	0.005 [0.195 – 0.817]	0.298 [-0.616 0.228]	0.861 [-0.499 – 0.431]	0.126 [-0.683 0.116]	0.077 [-0.738 0.062]
**Pancreas PDFF (average)**	0.426	0.210	0.487*	0.365	0.453*	0.021	-0.008	-0.214	-0.312
p-value [CI]	0.054 [-0.021 - 0.731]	0.360 [-0.256 - 0.598]	0.025 [0.056 – 0.765]	0.104 [-0.093 - 0.695]	0.039 [0.013 – 0.746]	0.929 [-0.426 0.459]	0.974 [-0.472 0.459]	0.352 [-0.600 0.253]	0.193 [-0.679 0.179]
**Pancreas PDFF (tail)**	0.235	0.155	0.558*	0.510*	0.597*	0.263	0.020	-0.051	0.145
p-value [CI]	0.305 [-0.232 - 0.614]	0.502 [-0.309 - 0.560]	0.009 [0.154 – 0.803]	0.018 [0.086 – 0.777]	0.004 [0.210 – 0.823]	0.249 [-0.203 0.632]	0.934 [-0.450 0.481]	0.827 [-0.483 0.401]	0.554 [-0.344 0.572]
**Pancreas PDFF (body)**	0.296	0.134	0.455*	0.397	0.473*	0.046	0.077	-0.207	0.089
p-value [CI]	0.192 [-0.169 - 0.653]	0.563 [-0.328 - 0.544]	0.038 [0.015 – 0.747]	0.075 [-0.055 - 0.714]	0.030 [0.038 – 0.757]	0.845 [-0.405 0.479]	0.755 [-0.403 0.524]	0.369 [-0.595 0.260]	0.716 [-0.393 0.533]
**Pancreas PDFF (head)**	0.555*	0.204	0.384	0.280	0.304	0.328	-0.077	0.234	.032
p-value [CI]	0.009 [0.148 - 0.801]	0.375 [-0.262 - 0.593]	0.085 [-0.070 - 0.707]	0.218 [-0.185 - 0.643]	0.180 [-0.160 - 0.658]	0.146 [-0.134 0.673]	0.755 [-0.524 0.403]	0.307 [-0.613 0.233]	0.898 [-0.441 0.490]
**Total fat area**	-0.158	0.208	0.725*	0.821*	0.783*	0.105	0.136	-0.188	-0.304
p-value [CI]	0.506 [-0.571 - 0.319]	0.378 [-0.271 - 0.605]	0.001 [0.404 – 0.887]	0.001 [0.586 – 0.929]	0.001 [0.512 – 0.913]	0.658 [-0.366 0.534]	0.591 [-0.367 0.577]	0.427 [-0.591 0.290]	0.219 [-0.683 0.204]
**Visceral fat area (VAT)**	0.107	0.079	0.441	0.579*	0.543*	0.382	0.122	-0.059	-0.094
p-value [CI]	0.654 [-0.365 - 0.535]	0.741 [-0.389 - 0.514]	0.052 [-0.016 - 0.745]	0.007 [0.170 – 0.818]	0.013 [0.118 – 0.800]	0.096 [-0.086 0.713]	0.629 [-0.378 0.568]	0.803 [-0.500 0.405]	0.711 [-0.548 0.403]
**Retroperitoneal fat area**	0.099	0.083	0.313	0.390	0.292	0.293	0.201	-0.179	-0.247
p-value [CI]	0.677 [-0.371 - 0.529]	0.729 [-0.385 - 0.517]	0.179 [-0.164 - 0.671]	0.089 [-0.077 - 0.717]	0.212 [-0.187 - 0.658]	0.210 [-0.186 0.659]	0.424 [-0.307 0.620]	0.450 [-0.585 0.299]	0.324 [-0.649 0.263]
**Intraperitoneal fat area**	0.119	0.057	0.477*	0.654*	0.588*	0.273	0.064	.008	-0.065
p-value [CI]	0.618 [-0.354 - 0.543]	0.811 [-0.407 - 0.498]	0.034 [0.029 – 0.765]	0.002 [0.285 – 0.854]	0.006 [0.183 – 0.822]	0.244 [-0.206 0.647]	0.800 [-0.427 0.527]	0.972 [-0.447 0.460]	0.798 [-0.527 0.427]
**Subcutaneous area (SAT)**	-0.284	0.219	0.704*	0.766*	0.758*	0.001	0.091	-0.166	-0.271
p-value	0.225	0.354	0.001	0.001	0.001	0.997	0.719	0.483	0.276

[CI]	[-0.654 - 0.195]	[-0.261 - 0.612]	[0.367 – 0.877]	[0.479 – 0.905]	[0.464 – 0.902]	[-0.453 0.454]	[-0.405 0.546]	[-0.577 0.311]	[-0.664 0.238]
**Thickness VAT**	0.068	-0.035	0.447*	0.612*	0.485*	0.197	0.221	-0.059	0.108
p-value<br>[CI]	0.774<br>[-0.398 - 0.506]	0.882<br>[-0.481 - 0.425]	0.048<br>[-0.009 - 0.749]	0.004<br>[0.219 – 0.834]	0.030<br>[0.040 – 0.770]	0.406<br>[-0.282 0.597]	0.379<br>[-0.288 0.632]	0.803<br>[-0.500 0.405]	0.669<br>-0.390 0.558
**Thickness SAT**	-0.195	0.093	0.338	0.438	0.313	-0.226	-0.019	-0.166	-0.092
p-value<br>[CI]	0.410<br>[-0.596 - 0.284]	0.698<br>[-0.377 - 0.524]	0.144<br>[-0.136 - 0.687]	0.053<br>[-0.019 - 0.744]	0.179<br>[-0.164 - 0.671]	0.337<br>[-0.617 0.253]	0.942<br>[-0.493 0.464]	0.483<br>[-0.577 0.311]	0.717<br>[-0.546 0.404]
**IMT**	-0.056	-0.072	-0.101	-0.209	-0.165	0.070	0.221	-0.056	-0.089
p-value<br>[CI]	0.798<br>[−0.468 – 0.375]	0.746<br>[−0.480 – 0.362]	0.647<br>[−0.502 – 0.336]	0.339<br>[−0.581 – 0.235]	0.451<br>[−0.550 – 0.277]	[0.750<br>-0.363 0.479]	[0.335<br>-0.246 0.605]	0.798<br>[-0.468 0.375]	0.703<br>[-0.511 0.369]
**IMT/diameter**	0.125	-0.108	-0.058	-0.140	-0.155	0.008	0.202	-0.047	-0.075
p-value<br>[CI]	0.568<br>[−0.314 – 0.521]	0.623<br>[−0.508 – 0.330]	0.792<br>[−0.470 – 0.374]	0.523<br>[−0.532 – 0.300]	0.480<br>[−0.542 – 0.287]	0.971<br>[-0.416 0.429]	0.379<br>[-0.264 0.592]	0.831<br>[-0.461 0.384]	0.746<br>[-0.501 0.380]
**CAT**	-0.075	0.124	-0.159	-0.282	-0.264	-.240	-0.023	-0.191	-0.205
p-value<br>[CI]	0.740<br>[−0.492 – 0.369]	0.582<br>[−0.326 – 0.528]	0.481<br>[−0.553 – 0.294]	0.204<br>[−0.637 – 0.172]	0.236<br>[−0.625 – 0.191]	0.283<br>[-0.609 0.215]	0.922<br>[-0.472 0.435]	0.396<br>[-0.576 0.264]	0.387<br>[-0.602 0.275]
**CAS**	-0.229	-0.087	-0.276	-0.264	-0.311	-0.186	-.075	-0.060	-0.015
p-value<br>[CI]	0.306<br>[−0.602 – 0.226]	0.700<br>[−0.501 – 0.359]	0.214<br>[−0.633 – 0.178]	0.235<br>[−0.625 – 0.190]	0.159<br>[−0.655 – 0.140]	0.407<br>[-0.572 0.268]	0.754<br>[-0.511 0.392]	0.789<br>[-0.480 0.382]	0.950<br>[-0.466 0.442]
**CAS/CAT**	-.119	-.177	-.093	.016	.021	0.136	0.069	0.223	0.296
p-value<br>[CI]	0.597<br>[−0.525 – 0.330]	0.431<br>[−0.566 – 0.277]	0.680<br>[−0.505 – 0.354]	0.944<br>[−0.420 – 0.445]	0.926<br>[−0.415 – 0.449]	0.547<br>[-0.315 0.537]	0.771<br>[-0.397 0.507]	0.318<br>[-0.232 0.598]	0.205<br>[-0.182 0.661]
**PFT**	-0.049	0.021	0.363	0.410	0.452*	0.201	0.020	0.160	0.063
p-value<br>[CI]	0.828<br>[−0.472 – 0.392]	0.924<br>[−0.415 – 0.450]	0.097<br>[−0.082 – 0.688]	0.058<br>[−0.027 – 0.716]	0.035<br>[0.025 – 0.740]	0.371<br>[-0.254 0.583]	0.935<br>[-0.438 0.469]	0.477<br>[-0.293 0.554]	0.791<br>[-0.402 0.503]
**Peri-coronary fat**	-0.187	-0.168	0.242	0.362	0.313	0.097	-0.109	0.210	0.192
p-value<br>[CI]	0.406<br>[−0.573 – 0.267]	0.455<br>[−0.560 – 0.285]	0.278<br>[−0.213 – 0.611]	0.098<br>[−0.083 – 0.687]	0.156<br>[−0.138 – 0.656]	0.666<br>[-0.350 0.508]	0.648<br>[-0.536 0.363]	0.348<br>[-0.245 0.589]	0.417<br>[-0.287 0.594]
**Peri-apical fat**	0.407	0.197	0.415	0.432*	0.411	0.213	-0.094	-0.003	-0.129
p-value<br>[CI]	0.060<br>[−0.031 – 0.714]	0.379<br>[−0.257 – 0.580]	0.055<br>[−0.021 – 0.719]	0.045<br>[−0.001 – 0.728]	0.058<br>[−0.027 – 0.716]	0.342<br>[-0.242 0.591]	0.693<br>[-0.526 0.376]	0.990<br>[-0.435 0.430]	0.587<br>[-0.551 0.345]
**HOMA-IR**	0.090	0.368	0.686*	0.668*	0.759*	0.104	0.475	-0.100	0.000
p-value<br>[CI]	0.705<br>[−0.379 – 0.523]	0.111<br>[−0.103 – 0.704]	0.001<br>[0.337 – 0.869]	0.001<br>[0.307 – 0.861]	0.001<br>[0.466 – 0.902]	0.663<br>[-0.367 0.533]	0.034<br>[0.027 0.764]	0.674<br>[-0.530 0.370]	1.000<br>[-0.454 0.454]

TG index: triglyceride-glucose index
*Statistically significant results (p<0.05)

Table S4. Kruskall Wallis test for origin and Tanner stage, and Mann Whitney U test for sex, to compare with all liver, pancreas, abdominal fat compartment, and cardiovascular measurements (N=23).

	**Origin**	**Tanner stage**	**Sex**
	Kruskall Wallis		Mann Whitney
**Liver volume**	1.20	4.53	U= 36.00<br>Z= -0.330
p-value	0.75	0.21	0.78
**Liver PDFF**	6.67	4.93	U= 19.00<br>Z=-1.73
p-value	0.08	0.18	0.09
**MRS**	7.11	4.58	U= 30.00<br>Z= -0.979

p-value	0.07	0.33	0.36
**Pancreas volume**	3.83	8.95*	U= 32.00<br>Z= -0.661
p-value	0.28	0.030	0.55
**Pancreas PDFF (average)**	2.00	7.62	U= 32.00<br>Z= -0.661
p-value	0.57	0.054	0.55
**Pancreas PDFF (tail)**	3.50	3.35	U= 37.00<br>Z= -0.248
p-value	0.32	0.34	0.84
**Pancreas PDFF (body)**	3.96	5.06	U= 34.00<br>Z= -0.495
p-value	0.27	0.17	0.66
**Pancreas PDFF (head)**	0.50	10.47*	U= 31.00<br>Z= -0.743
p-value	0.92	0.015	0.50
**Total fat area**	4.77	1.17	U= 36.00<br>Z= -0.131
p-value	0.19	0.76	0.93
**Visceral fat area (VAT)**	4.96	3.35	U= 37.00<br>Z= -0.044
p-value	0.18	0.34	1.00
**Retroperitoneal fat area**	0.96	2.15	U= 37.00<br>Z= -0.044
p-value	0.81	0.54	1.00
**Intraperitoneal fat area**	4.35	3.09	U= 37.00<br>Z= -0.044
p-value	0.23	0.38	1.00
**Subcutaneous area (SAT)**	4.12	0.60	U= 35.00<br>Z=-0.218
p-value	0.25	0.90	0.87
**Thickness VAT**	3.02	8.00*	U= 34.00<br>Z= -0.306
p-value	0.39	0.046	0.80
**Thickness SAT**	6.14	4.38	U= 33.00<br>Z= -0.393
p-value	0.10	0.22	0.74
**IMT**	1.810	0.83	U= 42.50<br>Z= -0.187
p-value	0.61	0.93	0.86
**IMT/ diameter**	1.01	3.54	U= 41.00<br>Z= -0.298
p-value	0.80	0.47	0.80
**CAT**	1.60	8.32	U= 39.00<br>Z= -0.274
p-value	0.66	0.08	0.82
**CAS**	2.89	4.85	U= 39.00<br>Z= -0.274
p-value	0.41	0.30	0.82
**CAS/CAT**	3.65	5.20	U= 40.00<br>Z= -0.196
p-value	0.30	0.27	0.88
**PFT**	2.10	3.06	U= 35.00<br>Z= -0.588
p-value	0.55	0.38	0.60
**Peri-coronary fat**	0.89	4.74	U= 27.50<br>Z= -1.177
p-value	0.83	0.19	0.25
**Peri-apical fat**	0.45	2.44	U= 40.00<br>Z= -0.196

p-value	0.93	0.49	0.88
**HOMA-IR**	5.22	3.83	U= 21.00<br>Z= -1.440
p-value	0.16	0.43	0.17

*On average, participants with Tanner stage 5 had the highest pancreas volume and pancreas head PDFF, while those with Tanner stage 4 had higher VAT thickness.
